# Management of mass casualty incidents: a systematic review and clinical practice guideline update

**DOI:** 10.1007/s00068-024-02727-0

**Published:** 2025-01-10

**Authors:** Arnold J. Suda, Axel Franke, Miriam Hertwig, Käthe Gooßen

**Affiliations:** 1 Centre for Orthopaedics and Trauma Surgery, University Medical Centre Mannheim, Medical Faculty Mannheim of Heidelberg University, Theodor-Kutzer-Ufer 1-3, 67168 Mannheim, Germany; 2 Department for Trauma Surgery and Orthopaedics, Reconstructive and Septic Surgery, Sportstraumatology, German Armed Forces Hospital Ulm, Oberer Eselsberg 40, 89081 Ulm, Germany; 3 Institute for Research in Operative Medicine (IFOM), Faculty of Health, School of Medicine, Witten/Herdecke University, Ostmerheimer Str. 200, 51109 Cologne, Germany

**Keywords:** Mass casualty incidents, Polytrauma, Guideline, Triage, MANV

## Abstract

**Purpose:**

Our aim was to generate evidence- and consensus-based recommendations for the management of mass casualty incidents (MCIs) based on current evidence. This guideline topic is part of the 2022 update of the German guideline on the treatment of patients with severe/multiple injuries.

**Methods:**

MEDLINE and Embase were systematically searched to August 2021. Further literature reports were obtained from clinical experts. Randomised controlled trials, cross-sectional studies, prospective cohort studies, and comparative registry studies were included if they compared triage algorithms, interventions for MCI training, logistics or transport, decontamination, diagnosis or therapy during MCIs in the prehospital and hospital settings. We considered patient-relevant clinical outcomes such as mortality, diagnostic outcomes including sensitivity and specificity, rates of undertriage and overtriage as well as resource use. Risk of bias was assessed using NICE 2012 checklists. The evidence was synthesised narratively, and expert consensus was used to develop recommendations and determine their strength. Population, intervention, comparison, and outcome (PICO) questions from clinical questions were developed by clinical experts and guideline methodologists.

**Results:**

We screened 321 records in the original guideline version and 4225 during this update. Twenty-five studies were included, all of them from the updated search from 2009 to 2021. Twenty-five new studies were identified. Interventions covered were triage training (*n* = 7 studies), prehospital triage (*n* = 6), secondary triage (*n* = 2), transport/logistics (*n* = 3), decontamination (*n* = 5), and therapy (*n* = 2) during MCIs. Three new recommendations were developed. All achieved strong consensus.

**Conclusion:**

Due to unsatisfactory evidence, recommendations could only be made on training for improving triage quality and regular exercises for testing a hospital’s emergency response plan. No triage algorithm can be scientifically proven to be superior in all aspects. The key recommendation is the following: To improve triage quality, exercises or (virtual) training should be conducted in-house using verified triage systems and algorithms.

## Introduction

A mass casualty incident (MCI) is an emergency that is associated with a large number of people who have sustained injuries or damage. Such an incident requires special preparatory planning and organizational measures because it cannot be managed with existing and deployable prehospital and hospital resources that are available or are kept readily available. An MCI must be distinguished from a disaster, which—as defined in German Institute for Standardisation DIN 13050:2021-10 (Emergency Medical Services—Terms and Definitions)—is an incident that exceeds the severity of a mass casualty incident. It causes massive disruption and damage to local infrastructure and cannot be medically managed with emergency medical services (EMS) resources and operational structures alone [1]. A mass casualty incident with a large number of injured patients does not necessarily fulfil the legal criteria of a disaster situation (e.g. flooding). For this reason, different approaches are used to manage these scenarios.

A mass casualty incident with a large number of severely injured patients is a challenge for EMS personnel at the scene and for the receiving hospitals. Triage is performed to ensure that available personnel and material resources are used in such a way that individualised medical care can be provided to casualties with maximum efficiency. Tactical considerations may require that care providers depart from the principles of individualised medical care in an attempt to increase the number of survivors. This approach presents a major challenge and burden for all care providers involved in the process and requires particular attention and training. Studies have shown that both prehospital and inhospital exercises can improve triage quality [2–4]. Virtual reality training is a useful option, too [5–8]. The studies published by Risavi et al. in 2013 [4] and by Mills et al. in 2020 [7] are particularly interesting in this context but show a few weaknesses in study design, such as a small number of participants and selection bias.

The need to develop appropriate recommendations has been recognised and addressed in the previous version of this guideline. The objective of this work was to update the existing guideline with the current literature, which is done regularly every five years.

## Methods

This guideline topic is part of the 2022 update of the German guideline on the treatment of patients with severe/multiple injuries [31]. The guideline update is reported according to the RIGHT tool [32], the systematic review part according to the Preferred Reporting Items for Systematic Reviews and Meta-Analyses (PRISMA) 2020 reporting guideline [33]. The development of recommendations followed standard methodology set out in the guideline development handbook by the Association of the Scientific Medical Societies in Germany (AWMF) [34]. All methods were defined a priori, following the methods report of the previous guideline version from July 2016 [35] with minor modifications, as detailed below. The introduction and discussion sections of this publication are translated from an abridged version of the original guideline text [31].

### PICO questions and eligibility criteria

Clinical experts and guideline methodologists developed population, intervention, comparison, and outcome (PICO) questions from clinical questions. The process was informed by a pilot literature search. In addition, the participating professional societies involved in guideline development were asked to submit new PICO questions. The overarching PICO question for this topic area was:

*In the context of mass casualty incidents, does prehospital or clinical MCI triage, triage training for medical staff, or MCI-specific logistics, transport, diagnosis, or therapy improve patient-relevant outcomes compared to any other intervention?*

The full set of pre-defined PICO questions is listed in Table S1 (Online Resource). The study selection criteria in the PICO format are shown in Table 1*.*

**Table 1:** Pre-defined selection criteria

Population:	• All injured patients in a real or simulated mass casualty incident, with patient data from prospective comparative studies or comparative registry studies (triage algorithms) *or* • Medically trained personnel (training, logistics or transport interventions)* or* • Left open (diagnostic or therapeutic interventions, decontamination)
Intervention /comparison:	• Primary (prehospital) and secondary (in-hospital) triage methods and algorithms • Training, logistics or transport interventions • Diagnostic or therapeutic interventions, decontamination; each in an MCI context
Outcomes:	• Sensitivity, specificity, AUC, % correct triage, rate of overtriage and undertriage, triage time (triage algorithms, triage training) • Time or personnel resources, utilisation/over-utilisation rates, patient-relevant outcomes (logistics or transport) • Sensitivity, specificity, PPV, NPV, AUC, time to result (diagnostic interventions) • Duration or success of intervention, patient-relevant outcomes (therapeutic interventions, decontamination)
Study type:	• Comparative, prospective studies (randomised controlled trials, cohort studies) • Cross-sectional studies (for triage/diagnostic interventions) • Comparative registry^a^ data (incl. case–control studies) • Systematic reviews based on the above primary study types
Language:	English or German
Other inclusion criteria:	• Full text of study published and accessible • Study matches pre-defined PICO question
Exclusion criteria:	• Studies with data from non-trauma patients, with simulated patient data, studies on cadavers or cells • Multiple publications of the same study without additional information

^a^Using the Agency for Healthcare Research and Quality's (AHRQ) definition of registries [36]

### Literature search

An information specialist systematically searched for literature in MEDLINE (Ovid) and Embase (Elsevier). The search strategy was developed based on the PICO questions. It contained index (MeSH/Emtree) and free text terms for the population and intervention. All searches were completed on 27 August 2021. The start date for the search was 1 January 2009. Table S2 (Online Resource) provides details for all searches. Clinical experts were asked to submit additional relevant references.

### Study selection

Study selection was performed independently by two reviewers in a two-step process using the predefined eligibility criteria: (1) title/abstract screening of all references retrieved from the database searches using the Rayyan software [36] and (2) full-text screening of all articles deemed potentially relevant by at least one reviewer at the title/abstract level in Endnote (Endnote, Version: 20 [Software]. Clarivate, Boston, Massachusetts, USA. https://endnote.com/). Disagreements were resolved through consensus or by consulting a third reviewer. The reasons for full-text exclusion were recorded (Table S3, Online Resource).

### Assessment of risk of bias and level of evidence

Two reviewers sequentially assessed the risk of bias of included studies at study level using the relevant checklists from the NICE guidelines manual 2012 [37], and assigned each study an initial level of evidence (LoE) using the Oxford Centre for Evidence-based Medicine Levels of Evidence (2009) [38]. For studies with baseline imbalance and unadjusted analyses, post-hoc secondary analyses, indirectness of the study population, or low power and imprecision of the effect estimate, the LoE was downgraded and marked with an arrow (↓). Any disagreements were resolved through consensus or by consulting a third reviewer.

### Data extraction and data items

Data were extracted into a standardised data table by one reviewer and checked by another. A predefined data set was collected for each study, consisting of study characteristics (study type, aims, setting), participant selection criteria and baseline characteristics (age, gender, injury scores, other relevant variables), intervention and control group treatments (including important co-interventions), patient flow (number of patients included and analysed), matching/adjusting variables, and data on outcomes for any time point reported.

### Outcome measures

Outcomes were extracted as reported in the study publications. For prospective cohort studies and registry data, preference was given to data obtained after propensity-score matching or statistical adjustment for risk-modulating variables over unadjusted data.

### Synthesis of studies

Studies were grouped by interventions. An interdisciplinary expert group used their clinical experience to synthesise studies narratively by balancing beneficial and adverse effects extracted from the available evidence. Priority was given to reducing mortality and triage/diagnostic accuracy. Clinical heterogeneity was explored by comparing inclusion criteria and patient characteristics at baseline as well as clinical differences in the interventions and co-interventions.

### Development and updating of recommendations

For each PICO question, the following updating options were available: (1) the recommendation of the preceding version remains valid and requires no changes (“confirmed”); (2) the recommendation requires modification (“modified”); (3) the recommendation is no longer valid or required and is deleted; (4) a new recommendation needs to be developed (“new”). An interdisciplinary expert group of clinicians with expertise in Trauma and Military Surgery reviewed the body of evidence, drafted recommendations based on the homogeneity of clinical characteristics and outcomes, the balance between benefits and harms as well as their clinical expertise, and proposed grades of recommendation (Table 2). In the absence of eligible evidence, good practice recommendations were made based on clinical experience, data from studies with a lower level of evidence, and expert consensus in cases where the guideline group felt a statement was required due to the importance of the topic. These were not graded, and instead labelled as good (clinical) practice points (GPP). For GPPs, the strength of the recommendation was conveyed with the language used, as shown in Table 2.

**Table 2:** Three-level scheme for grading recommendations

Symbol	Grade of recommendation	Description	Language used (examples)
⇑⇑	A	Strong recommendation	“use …”, “do not use …”
⇑	B	Recommendation	“should use …”, “should not use …”
⇔	0	Open recommendation	“consider using …”, “… can be considered”

### Consensus process

The guideline group finalised the recommendations during a web-based, structured consensus conference on 14 June 2021 via the Zoom software (Zoom, Version: 5.x [Software]. Zoom Video Communications, Inc., San José, California, USA. https://zoom.us). A neutral moderator facilitated the consensus conference. Voting members of the guideline group were delegates of all participating professional organisations, including clinicians, emergency medical services personnel, and nurses, while guideline methodologists attended in a supporting role. Members with a moderate, thematically relevant conflict of interest abstained from voting on recommendations, members with a high, relevant conflict of interest were not permitted to vote or participate in the discussion. Attempts to recruit patient representatives were unsuccessful. A member of the expert group presented recommendations. Following discussion, the guideline group refined the wording of the recommendations and modified the grade of recommendation as needed. Agreement with both the wording and grade of recommendation was assessed by anonymous online voting using the survey function of Zoom. Abstentions were subtracted from the denominator of the agreement rate. Consensus strength was classified as shown in Table 3.

**Table 3:** Classification of consensus strength

Description	Agreement rate
Strong consensus	> 95% of participants
Consensus	> 75 to 95% of participants
Majority approval	> 50 to 75% of participants
No approval	< 50% of participants

Recommendations were accepted if they reached consensus or strong consensus. For consensus recommendations with ≤ 95% agreement, diverging views by members of the guideline group were detailed in the background texts. Recommendations with majority approval were returned to the expert group for revision and further discussion at a subsequent consensus conference. Recommendations without approval were considered rejected.

### External review

During a four-week consultation phase, the recommendations and background texts were submitted to all participating professional organisations for review. Comments were collected using a structured review form. The results were then assessed, discussed and incorporated into the text by the guideline coordinator with the relevant author group.

The guideline was adopted by the executive board of the German Trauma Society on 17 January 2023.

### Quality assurance

The guideline recommendations were reviewed for consistency between guideline topic areas by the steering group. Where necessary, changes were made in collaboration with the clinical leads for all topic areas concerned. The final guideline document was checked for errors by the guideline chair and methodologist.

## Results

**Fig. 1.**
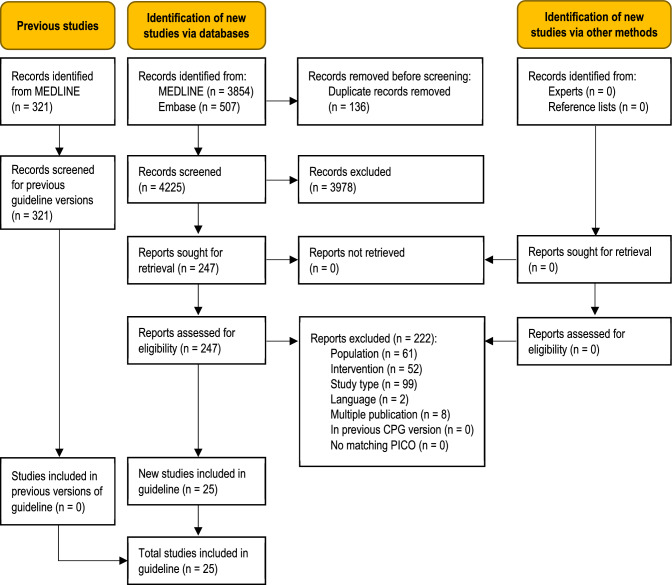
Modified PRISMA 2020 flow diagram showing the systematic literature search and selection of studies

The database searches identified 4225 unique records (Fig. 1). No additional records were obtained from clinical experts or from the reference lists of included studies. Twenty-five studies were eligible for this update [2–8, 10–17, 20, 24–28, 39–42]. None of the studies had been included in the previous version of this guideline topic. A total of 222 full-text articles were excluded (Table S3, Online Resource).

### Characteristics of studies included in this update

Study characteristics, main outcomes, levels of evidence, and risk-of-bias assessments are presented in Table 4. Full details are provided in Table S4 (Online Resource). This update included six RCTs [5, 8, 25, 26, 40, 42], four randomised cross-over studies [7, 24, 27, 41], six prospective cohort studies [2, 3, 6, 10, 24, 28], two prospective cross-over studies [4, 39] as well as eight cross-sectional studies [11–17, 20]. Ten studies were performed in Europe [2, 3, 6, 12, 13, 24–28], eight in North America [4, 5, 8, 11, 15, 16, 39, 40], three in Asia [17, 41, 42], two in Australia [7, 10], and two in a military setting [14, 20]. Eligible populations were either trauma victims (triage studies, pain management), medical personnel (training, logistics and transport studies, airway management), or adult volunteers (decontamination).

**Table 4:** Characteristics of studies included in the update (see Table S4, Online Resource for details)

Study, Reference	Population	Interventions (N participants analysed^a^)	Main outcome	LoE, risk of bias^b^, Comments
*Triage training for medical professionals*				
Andreatta 2010 [5] RCT	Post-graduate medical students	VR: full immersion virtual-reality training (N = 7) SP: standard simulated patient disaster drill (N = 8)	Triage performance rating, mean ± SD (5-point scale, 5 = best, 1 = worst) VR: 3.55 ± 0.17 SP: 3.47 ± 0.41	LoE: 2b↓ unclear RoB small sample size (imprecision)
Cicero 2017 [8] RCT	EMS providers (paramedics, paramedic students)	IG: weekly video game-based triage training for 13w between two disaster drills (N = 26) CG: no triage training between the drills (N = 21)	Triage accuracy at time 2 [%], median (IQR) IG: 90 (80 to 90) CG: 80 (70 to 80)	LoE: 2b↓ high risk of performance / attrition bias study underpowered (imprecision)
Dittmar 2016 [2] Prospective cohort study	ASAV-trained EMS professionals	IN: initial assessment after ASAV training (N = 76) FU: follow-up assessment ≥ 12 months after initial testing (N = 22)	Triage accuracy, % (95% CI) proportion of cases classified as intended IN: 84 (81–87) FU: 77 (70–85), p = 0.028	LoE: 2b unclear RoB substantially fewer participants at FU
Dittmar 2018 [3] Prospective cohort study, follow up of Dittmar 2016	EMS personnel who participated in prior study	IN: initial triage assessment (N = 51) FU: follow up assessment after a mean of 14.6 (14.0–15.3) m (N = 22) FU2: follow up assessment after a mean of 25.1 (22.9–27.3) min. + re-training (N = 19)	Triage accuracy, % (95% CI) IN: 84 (80–87) FU: 77 (69–85) 3rd: 86 (82–91) IN vs. FU: p = 0.159 FU2 vs. IN: p = 1.000 FU vs. FU2: p = 0.069	LoE: 2b high risk of attrition bias differential attrition (no volunteers at FU2)
Knight 2010 [6] Prospective cohort study	MIMMS course attendees (doctors, nurses, paramedics)	TT: triage trainer video game (N = 47) CS: card-sort exercise (N = 44) triage ability assessed in a simulated mass casualty situation afterwards	Tagging accuracy, n (%) n participants who correctly tagged 8/8 victims TT: 34 (72), p = 0.02 CS: 24 (55)	LoE: 3b↓ high risk of selection / performance bias unusual analysis of triage accuracy; very small number of casualties; direct financial conflict of interest
Mills 2020 [7] Randomised cross-over study	3rd year paramedical science students	VR: virtual reality MCI simulation (completed first by N = 15, second by N = 14) LS: live MCI simulation (completed first by N = 14, second by N = 15)	Cards allocated correctly [n], mean ± SD VR: 7.97 ± 1.81, p = 1.00 LS: 8.52 ± 1.66	LoE: 2b↓ low RoB very small number of casualties; direct financial conflict of interest
Risavi 2013 [4] Prospective cross-over study	Emergency medical technicians /paramedics	WR: written scenario triage exercise (completed first by N = 28, second by N = 17) MG: moulage scenario triage exercise (completed first by N = 17, second by N = 28)	Triage accuracy for red level, n/N (%) WR: 166/180 (92.2) MG: 163/180 (90.5) Triage accuracy for yellow level, n/N (%) WR: 182/315 (57.7) MG: 141/315 (44.7)	LoE: 2b low RoB small number of casualties
*Prehospital triage*				
Cicero 2021 [16] Cross-sectional study	Paediatric trauma victims, age 0–15y	N = 31,093 patients evaluated using each algorithm START: Simple triage and rapid treatment algorithm/Jump-START for patients under 9y CareFlight: CareFlight Australia algorithm FDNY: Fire department New York algorithm Reference: COT expected triage level	*COT level red (N* = *1,572)* Sensitivity, % (95% CI) START: 54 (51–56) CareFlight: 50 (48–53) FDNY: 56 (54–59) Specificity, % (95% CI) START: 85 (84–86) CareFlight: 89 (89–90) FDNY: 80 (79–80) *COT level yellow (N* = *11,587)* Sensitivity, % (95% CI) START: 21 (20–22) CareFlight: 22 (21–23) FDNY: 20 (19–21) Specificity, % (95% CI) START: 88 (87–89) CareFlight: 87 (87–88) FDNY: 88 (87–88)	LoE: 2b high risk of bias for patient selection / index tests no MCI setting
Cross 2013 [15] Cross-sectional study	Trauma victims	N = 530,695 patients evaluated using each algorithm IG1: START IG2: FDNY IG3: CareFlight IG4: GCS IG5: Sacco IG6: Unadjusted Sacco	Prognosis of mortality at hospital disposition, AUC (95% CI) START: 0.85 (0.84–0.85) FDNY: 0.85 (0.85–0.85) CareFlight: 0.85 (0.85–0.86) GCS: 0.825 (0.82–0.83) Sacco: 0.883 (0.88–0.89) Unadj. Sacco: 0.82 (0.82–0.83)	LoE: 2b no RoB tool prespecified^c^ no MCI setting
Cross 2015 [11] Cross-sectional study	Trauma victims	530,695 patients evaluated using each triage algorithm StartOver60: category changed from green to yellow for patients aged > 60 (N = 91,163) StartOver75: changed from green to yellow for patients aged > 75 (N = 53,195) Reference: START triage	Prognosis of mortality at hospital discharge, AUC START: 0.846 StartOver60: 0.855* StartOver75: 0.858* * 95% CI do not overlap with the reference method (START)	LoE: 2b no RoB tool prespecified^c^ (high risk of bias diagnostic outcomes); same population as in Cross 2013; no MCI setting
Malik 2020 [12] Cross-sectional study	Trauma victims	N = 195,709 patients evaluated using each algorithm BCD: Battlefield Casualty Drills CareFlight: CareFlight algorithm START: simple triage and rapid treatment algorithm JumpSTART: START algorithm for children < 8y mSTART: modified START MIMMS: Major Incident Medical Management and Support MPTT: Modified Physiological Triage Tool MPTT-24: Modified Physiological Triage Tool-24 NARU: National Ambulance and Resilience Unit Triage Sieve RAMP: Rapid Assessment of Mentation and Pulse Reference: intervention-based criteria	Prognosis of mortality, AUC (95% CI) *Age group 16–64y* BCD: 0.73 (0.72–0.74) CareFlight: 0.79 (0.78–0.80) START: 0.80 (0.79–0.81) JumpSTART: 0.77 (0.77–0.78) mSTART: 0.80 (0.79–0.81) MIMMS: 0.76 (0.75–0.77) MPTT: 0.46 (0.45–0.47) MPTT-24: 0.47 (0.46–0.48) NARU: 0.79 (0.78–0.79) RAMP: 0.70 (0.69–0.71) *Age group* ≥ *65y* BCD: 0.62 (0.61–0.62) CareFlight: 0.61 (0.60–0.61) START: 0.619 (0.62–0.62) JumpSTART: 0.57 (0.57–0.58) mSTART: 0.62 (0.62–0.63) MIMMS: 0.57 (0.57–0.58) MPTT: 0.58 (0.58–0.59) MPTT-24: 0.58 (0.58–0.59) NARU: 0.60 (0.60–0.60) RAMP: 0.60 (0.59–0.60)	LoE: 2b no RoB tool prespecified^c^ (low risk of bias for diagnostic outcomes) no MCI setting
Martin-Rodrigues 2019 [13] Cross-sectional study	Trauma patients (subgroup of full study set), attended by ALS and transferred to the ED	N = 262 patients evaluated using each algorithm SI: Shock Index GAP: Glasgow Age-Pressure Score RTS: Revised Trauma Score NEWS2: National Early Warning Score 2	Prognosis of 48 h-mortality, AUC (95% CI) SI: 0.48 (0.32–0.64) GAP: 0.98 (0.91–1.00) RTS: 0.96 (0.88–1.00) NEWS2: 0.96 (0.88–1.00)	LoE: 3b↓ no RoB tool prespecified^c^; no MCI setting; wide confidence intervals (imprecision)
Vassallo 2017 [14] Cross-sectional study	Trauma patients presenting to military ED	N = 3,645 patients evaluated using each algorithm MPTT: Modified Physiological Triage Tool MS: Military Sieve MMS: Modified Military Sieve TS: Triage Sieve START: Simple triage and rapid treatment CareFlight: CareFlight algorithm Reference: P1 status (one or more life-saving interventions from a predefined list)	Diagnosis of P1 status, sensitivity, % (95% CI) MPTT: 69.9 (67.7–72.0) MS: 43.8 (41.5–46.2) MMS: 50.9 (48.6–53.3) TS: 24.8 (22.8–26.9) START: 38.7 (36.5–41.1) CareFlight: 33.5 (31.3–35.8) Diagnosis of P1 status, specificity, % (95% CI) MPTT: 65.3 (63.2–67.5) MS: 93.6 (92.4–94.6) MMS: 87.5 (85.9–88.9) TS: 94.7 (93.6–95.7) START: 96.9 (96.0–97.6) CareFlight: 98.4 (97.7–98.9)	LoE: 2b high risk of bias for the index tests no MCI setting, military
*Secondary triage*				
Muguruma 2019 [17] Cross-sectional study	Trauma patients	N = 2,005 patients evaluated using each algorithm PPTAS: Pediatric Physiological and Anatomical Triage Score TRTS: Triage Revised Trauma Score Reference: need of immediate treatment, approximated by ICU admission	Diagnosis of ICU admission, AUC (95% CI) PPTAS: 0.61 (0.59–0.63) TRTS: 0.57 (0.56–0.59), p < 0.001	LoE: 2b unclear RoB no MCI setting
Vassallo 2015 [20] Cross-sectional study	Trauma patients presenting at military ED	482 patients evaluated using each algorithm TSO: Triage Sort SI: shock index, cut-off > 0.75 Reference: Priority 1 status (need of one or more life-saving interventions from a predefined list)	Diagnosis of P1 status, sensitivity, % (95% CI) TSO: 58.6 (51.8–65.4) SI: 70 (63.6–76.3) Diagnosis of P1 status, specificity, % (95% CI) TSO: 88.7 (83.5–93.9) SI: 74.7 (67.5–81.8)	LoE: 2b unclear RoB no MCI setting, military
*Logistics and transport*				
Cheng 2020 [40] Prospective cross-over study	Medical students with training in basic disaster life support	IG: Flags and Tags attached to the manikins (N = 44) CG: Tags only at the manikins (N = 38)	Completion time [s], MD (95% CI) IG: –24.42 (–21.11 to –27.73); 28.5% reduction Accuracy^§^, n/N (%) IG: 54 (64.3) vs. CG: 49 (58.3) ^§^Successfully identified all 10 reds	LoE: 2b high risk of performance bias simulated casualties
Cuttance 2017 [10] Prospective cohort study	Operational clinical staff	IG1: With aide-memoir while performing the exercise (N = 73) IG2: Educational refresher before performing the exercise, no aide-memoir (N = 74) IG3: Educational refresher before performing the exercise, with aide-memoir (N = 74) CG: No supporting documentation (N = 71)	Accuracy rate [%], mean ± SD^§^ IG1: 90 ± 6.7 IG2: 76 ± 6.6 IG3: 89 ± 7.3 CG: 47 ± 6.2 ^§^Numbers calculated from those in text p. 7 (by dividing by n in each group) to match those in abstract	LoE: 2b low RoB simulated casualties
Homier 2018 [41] RCT	ED staff	SMS: short message service (N = 44) IMA: instant messaging application (N = 44) CG: manual phone tree (N = 44)	Respondents at 45 min, n/N (%) SMS: 7/44 (16) IMA: 11/44 (25) CG: 18/44 (41), p = 0.029 Response time [min], median (range) SMS: 152 (2.0 to 336) IMA: 104 (1.0 to 458) CG: 8.5 (2.0 to 8.5)	LoE: 2b↓ unclear RoB wide confidence intervals (imprecision)
*Therapy*				
Ophir 2014 [42] Randomised cross-over study	Medical staff	LMAU: first-generation SAD laryngeal mask AW unique (n = 117, 6 procedures each) LTS-D: second-generation SAD laryngeal tube suction disposable (n = 117, 6 procedures each) SLMA: second-generation SAD supreme laryngeal mask AW (n = 117, 6 procedures each) CG: endotracheal tube size 8 with direct laryngoscopy (n = 117, 6 procedures each)	Procedure failure, n *(3 sequential unsuccessful attempts)* LMAU: 0 LTS-D: 0 SLMA: 0 CG: 9 Time to successful AW control [s], mean LMAU: 17.2, p < 0.0001 LTS-D: 18.1, p < 0.0001 SLMA: 17.7, p < 0.0001 CG: 31.7	LoE: 2b↓ unclear RoB casualties were manikins (indirectness)
Shimonovich 2016 [43] RCT	Mild to moderate blunt trauma patients^d^	IN KET: intranasal ketamine 1.0 mg/kg (n = 24) IV MO: intravenous morphine 0.10 mg/kg (n = 24) IM MO: muscle injection of morphine 0.15 (n = 27)	Time to onset [min], mean (95% CI) IN KET: 14.3 (9.8–18.8) IV MO: 8.9 (6.6–11.2), p = 0.300 IM MO: 26.0 (20.3–31.7), p = 0.003 Time to maximal pain reduction [min] (95% CI) IN KET: 40.4 (33.9–46.9) IV MO: 33.4 (26.2–40.6), p = 0.441 IM MO: 46.7 (41.1–52.3), p = 0.386 IV MO vs. IM MO: p = 0.019	LoE: 1b high risk of performance / attrition bias no severe trauma patients
*Decontamination*				
Amlot 2010 [25] RCT	Volunteers	IG1: standard UK protocol with 3 min. shower: 2-min. delivery of detergent solution followed by a water-only rinse for a further min. 37 °C and ~ 6,000 L h^−1^ (= flow of ~ 10 L min^−1^ per person; n = 15) IG2: 3 min. shower with washcloth (n = 15) IG3: 3 min. shower with pictorial instructions (n = 13) IG4: 3 min. shower with washcloth + instructions (n = 20) IG5: 6 min. shower (n = 11) CG: no decontamination (n = 15)	Relative efficacy, p-value % Efficacy = 100 – ((Qpre – Qpost)/Qpre) × 100, with Q: amount of fluorophore on the skin surface IG1: p < 0.05 IG2: p < 0.05 IG3: p < 0.05 IG4: p < 0.05 IG5: p < 0.05 CG: reference	LoE: 2b↓ unclear RoB simulated contamination, no patient-relevant outcomes (indirectness)
Amlot 2017 [24] *Study 1:* randomised cross-over study *Study 2:* prospective cohort study	Volunteers	*Study 1* (N = 20, 12 analysed) BR-B: dry decontamination with blue roll, blot the simulant application site (n = 5) BR-R: dry decontamination with incontinence pad, rub the simulant application site (n = 5) BR-BR: dry decontamination with blue roll, blot & rub (n = 5) IP-B: dry decontamination with incontinence pad, blot (n = 7) IP-R: dry decontamination with incontinence pad, rub (n = 7) IP-BR: dry decontamination with incontinence pad, blot & rub (n = 7) CG: no decontamination, control arm (all) *Study 2 (N* = *21)* IG: guidance during dry decontamination with blue roll (n = 10) CG: no guidance during dry decontamination with blue roll (n = 11)	*Study 1* Spread of simulant [cm^2^], MD ± SD BR-B: − 4.64 ± 9.03 BR-R: 4.23 ± 4.08 BR-BR: 2.17 ± 1.39 IP-B: 3.85 ± 11.98 IP-R: 7.77 ± 6.20 IP-BR: 2.06 ± 4.89 blot vs. rub vs. blot&rub: p = 0.06 blue roll vs. incontinence pad: p = 0.14 MeS recovered post decontamination (µg/mL] Blot vs. CG: p < 0.05 Rub vs. CG: p > 0.05 Blot&rub vs. CG: p < 0.05 blot vs. rub vs. blot&rub: p > 0.05 blue roll vs. incontinence pad: p > 0.05 *Study 2* Whole body decontamination success, n/N (%) IG: 10/10 (100) CG: 2/11 (18) Top-down decontamination success, n/N (%) IG: 7/10 (70) CG: 1/11 (9)	*Study 1* LoE: 2b↓ high risk of attrition bias simulated contamination, large standard deviations (imprecision), no patient-relevant outcomes (indirectness) *Study 2* LoE: 3b↓ unclear RoB simulated contamination, no patient-relevant outcomes (indirectness)
Chilcott 2019 [26] RCT	Volunteers	Study 1: Dry decontamination IG: disrobe and dry with Blue Roll decontamination (n = 8) CG: not decontaminated: disrobe and roll between the stretchers only (n = 8) Study 2: Wet decontamination IG: decontamination: 4-min. protocol for casualty decontamination, rinse & wash with absorbent cellulose sponges, roll, repeat; towelling after decontamination; swabbing (n = 9) CG: no decontamination (n = 9)	Study 1: Dry decontamination, unclothed Left cheek p = 0.0024 Right cheek p < 0.0001 Front neck p = 0.0005 Back neck p = 0.0249 Left palm p = 0.0003 Right palm p = 0.0003 Left foot p = 0.0010 Right foot p = 0.0007 Left hand p = 0.0016 Right hand p = 0.0088 Top head p = 0.1613 (n.s.) Back head p = 0.3598 (n.s.) Study 1: Dry decontamination, clothed Mid-torso, front p = 0.0758 (n.s.) Mid-torso, back p = 0.0350 Right elbow (front) p = 0.0464 Left elbow (back) p = 0.1524 (n.s.) Left shin p = 0.1615 (n.s.) Right calf p = 0.4282 (n.s.) Study 2: Wet decontamination, unclothed Left cheek p < 0.0001 Right cheek p < 0.0001 Front neck p < 0.0001 Back neck p = 0.0001 Left palm p < 0.0001 Right palm n.m Left foot p < 0.0001 Right foot p < 0.0001 Left hand n.m Right hand p < 0.0001 Top head p = 0.0070 Back head p = 0.0050	LoE: 2b↓ unclear RoB simulated contamination, no patient-relevant outcomes (indirectness)
Collins 2021 [27] Randomised cross-over study	Volunteers	IG1: improvised dry decontamination after 15 min. + interim (high-volume showering) after 25 min. (n = 11) IG2: improvised wet: ‘rinse wipe rinse’ after 15 min. + interim after 25 min. (n = 11) IG3: improvised dry after 15 min. + Interim after 25 min. + Specialist Operational Response procedure in a mass decontamination unit after 60 min. with structured showering (warm water, detergent and washing aids, n = 11) IG4: RWR after 15 min. + Interim after 25 min. + SOR after 60 min. (n = 11) CG: no decontamination (n = 11)	*MeS Decontamination* Decontamination efficacy, % % reduction in skin recovery vs. CG IG1: 93.1 IG2: 93.8 IG3: 92.8 IG4: 93.0; all p < 0.1 vs. CG *BeS Decontamination* Decontamination efficacy, % % reduction in skin recovery vs. CG IG1: 76.4 IG2: 83.5 IG3: 91.6 IG4: 81.2; all p < 0.001 vs. CG SOR vs. dry/wet + interim: p = 0.0189	LoE: 2b↓ low RoB simulated contamination, no patient-relevant outcomes (indirectness)
Larner 2020 [28] Prospective cohort study	Volunteers	DD: dry decontamination, using wound dressings 4 min. post dose (n = 11) LPS: ladder pipe system (showering corridor) 8 min. post dose, no towel (n = 10) TD: technical decontamination (decontamination tent) 12 min. post dose (n = 10) DD + LPS (n = 12) LPS + TD (n = 10) DD + TD (n = 12) LPS + towel: LPS + disposable cotton towel (n = 10) DD + LPS + towel (n = 10) DD + LPS + TD (no towel) (n = 10) DD + LPS + TD + towel (n = 10) Control: no decontamination (n = 10)	*Skin decontamination outcomes* MeS recovery, back swabs, p-value^s^ DD: n.s LPS: n.s TD: n.s DD + LPS: n.s LPS + TD: n.s DD + TD: p < 0.05 LPS + towel: n.s DD + LPS + towel: p < 0.05 DD + LPS + TD (no towel): p < 0.01 DD + LPS + TD + towel: p < 0.001 *Systemic exposure* no significant differences across groups	LoE: 2b unclear RoB simulated contamination

^*^Data are reported by IG vs. CG unless specified otherwise. ^a^Further details for participant flow provided in Table S4; ^b^risk of bias abbreviated as follows for the sake of this summary table: low RoB = RoB low for all domains; unclear RoB = RoB unclear for at least one domain, no high RoB in any domain; for studies with high RoB, all domains with high RoB are named, with RoB low or unclear for all other domains (for full details Table S4, Online Resource); ^c^no RoB tool available for prognostic studies within NICE 2012 checklists; ^d^GCS of 15, SBP 90–160, < 100 bpm. For abbreviations and acronyms, see the end of this guideline

### Risk-of-bias assessment for included studies and levels of evidence

The risk of bias was unclear for eleven studies that reported insufficient study details. Eight studies, all RCTs or secondary analyses of RCT data, were judged to be at low risk of bias in all domains. The risk of selection bias was high in six further studies, eleven were at high risk of performance bias, and two were at high risk of attrition bias.

The level of evidence was downgraded for 11 studies. Reasons for downgrading were low power and imprecision of the effect estimate (6 studies) as well as indirectness for five studies that included manikins instead of casualties or reported surrogate outcomes only.

### Recommendations

One new recommendation and two good practice points were developed based on the updated evidence and expert consensus (Table 5). All achieved strong consensus.

**Table 5:** List of recommendations with grade of recommendation and % consensus

No.	GoR	New evidence, Consensus	Recommendation	Status 2022
1	B ⇑	[2–8] 100%	To improve triage quality, exercises or (virtual) training should be conducted in-house using verified triage systems and algorithms	New
2	GPP	– 100%	Ensure that each hospital prepares a hospital emergency response plan, implements it In-house, and regularly evaluates it through exercises	New
3	GPP	– 100%	Physicians in charge should be prepared for a (terror) MCI scenario through regular exercises	New

*GoR* grade of recommendation, *GPP* good (clinical) practice point, *LoE* level of evidence, *MCI* mass casualty incident

## Discussion

### Rationale for recommendations

The current evidence base is still unsatisfactory. Scientific research findings only allow recommendations to be made on training for improving triage quality and regular exercises for testing a hospital’s emergency response plan.

The specific in-hospital organisational and medical adaptations that are necessary in order to be able to manage a mass casualty incident need to be precisely defined in the hospital emergency response plan and regularly evaluated. This includes patient assessment, triage, principles of care, communication, personnel requirements, and personnel employment. Overtriage, i.e. the categorisation of a non-critical casualty as a patient in critical condition, can consume important resources that are thus unavailable for the management of patients who would benefit more from these resources than the overtriaged patient. A direct relationship between overtriage, undertriage and mortality among critically injured casualties was reported [9]. In a study conducted in 2017, Cuttance et al. [10] showed that aide-memoires can be a helpful triage tool in this context. A variety of triage systems and algorithms are available. Only a few have been verified in comparative studies that involve severely injured casualties and meet the inclusion criteria for the S3 Guideline. Among them are the Simple Triage and Rapid Treatment (START) algorithm, the National Early Warning Score 2 (NEWS2), the Battlefield Casualty Drills (BCD) Triage Sieve, and the Modified Physiological Triage Tool (MPTT) [11–15]. Most of these are useful tools for appropriate and accurate triage. However, the evidence regarding the effectiveness of triage tools for paediatric patients is inconclusive [16, 17]. In 2015, Streckbein et al. [18] assessed twelve national and international triage protocols based on an evidence-based literature search. They concluded that there was no sufficient evidence demonstrating that any of these protocols were superior to the others in all respects and that no triage protocol is established on a national scale in Germany. In retrospective studies that were conducted by Cross et al. in 2015 and Malik et al. in 2020 and involved large numbers of subjects, START and BCD were found to be useful triage systems, although these studies show methodological weaknesses [11, 12]. In North America, the Simple Triage and Rapid Treatment (START) algorithm is widely used and enables even local initial responders to triage injured casualties in an effective manner [19]. Patients with acute life-threatening conditions are identified based on ABCD priorities. They are assigned to triage category I (RED) and must receive treatment immediately. Following a secondary triage process, patients who need surgery for an acute condition, such as thoracotomy or laparotomy for bleeding control or decompression for traumatic brain injury, are transported to the nearest appropriate hospital as soon as possible at the discretion of the chief emergency physician (or the on-site medical coordinator) [20]. At the beginning of triage, all ambulant casualties are assigned to triage category III (GREEN) and are asked to walk to the collection point for casualties with minor injuries. This approach takes into account that only a small proportion of the large number of casualties will present with acute life-threatening conditions and will need immediate care, which includes life-saving interventions that can be performed at the scene and, depending on available resources, rapid transport to acute surgical care. The START tool and the previously used triage algorithm were further developed at the national level, and a modified version of the START (mSTART) algorithm was published in 2006. The mSTART algorithm defines the type and scope of emergency treatment, time and consequences of secondary triage with regard to the urgency of emergency transport, and critical findings that help identify triage category II (YELLOW) patients [21]. In Germany, the mSTART algorithm was evaluated by Paul et al. [22]. In 2012, Ladehof et al. [23] adapted the mSTART tool to military and police settings and introduced the tacSTART algorithm, which includes a response to critical bleeding prior to triage and provides further details. This approach is of particular importance since life-saving interventions, e.g. application of a tourniquet for haemorrhage control and decompression of a tension pneumothorax, must be performed as rapidly as possible in acute life-threatening situations. For this reason, first responders should be appropriately trained and equipped for such interventions. A mass casualty incident that involves noxious substances and requires casualty decontamination is a special scenario. A number of studies were identified in which volunteers tested different decontamination algorithms [24–28]. There were no studies investigating a “real” scenario.

In 2014, the German Society for Disaster Medicine (Deutsche Gesellschaft für Katastrophenmedizin, DGKM) and the Federal Office of Civil Protection and Disaster Assistance (Bundesamt für Bevölkerungsschutz und Katastrophenhilfe, BBK) proposed a universal pre-triage system for surgical and medical (non-surgical) conditions [29]. The Primary Ranking for Initial Orientation in Emergency Medical Services (PRIOR), which is partly based on the ABCDE approach, assesses “conditions” rather than physiological parameters and is not fully compatible with mSTART and tacSTART. The use of triage systems should be included in local considerations regarding not only medical care principles but also the coordination of cooperation between emergency medical services and other emergency responders [30]. Where necessary, triage algorithms need to be adapted to local settings and should also be harmonised with available disaster management plans and similar tools.

Specific spatial and infrastructural conditions need to be addressed with a view to ensuring optimal preparedness. A uniformly standardised hospital emergency plan for all hospitals would not be reasonable since such a plan cannot address hospital-specific structural and personnel issues. By contrast, the implementation of a defined series of steps covering the entire process from patient assessment and triage to care seems to be useful and effective, with the main focus on the principles of categorising, prioritising, coordinating, and implementing. Apart from damage control surgery, tactical abbreviated surgical care (TASC) needs to be implemented. TASC depends on the situation, which determines the extent of care provided. It is essential that hospitals conduct regular exercises to identify and address any weaknesses in their hospital emergency response plan. (Online) training and simulation games can help personnel maintain acquired skills and gain proficiency.

Terrorist attacks are a special type of mass casualty incidents. Mass casualty terrorist incidents differ considerably from other MCIs in terms of tactical and strategic responses and medical care requirements since they are associated with different injury patterns and different time courses (several attacks may take place in different locations and at different times). In addition, they can involve a persistent threat also to EMS personnel.

Approaches to the management of MCIs should, therefore, cover possible terrorist attacks, shooter incidents, and other life-threatening situations and address not only blunt injuries but also penetrating injuries caused by automatic firearms and specific injuries resulting from the use of unconventional improvised explosive devices.

Because of their special nature, these multidimensional injuries are a particular challenge. In Germany, only Bundeswehr medical personnel with deployment experience can be expected to possess sufficient expertise to manage these injuries.

In summary, the current evidence base is insufficient to allow strong recommendations to be made on the basis of the literature available in this area. It is recommended, however, that regular exercises and training be conducted and evaluated. According to expert opinion, simulation games can play an important role in helping responders make the right decisions in real emergencies [43].

### Limitations of the guideline

Patients’ values and preferences were sought but not received. The effect of this on the guideline is unclear, and there is a lack of research evidence on the effect of patient participation on treatment decisions or outcomes in the emergency setting. A recent survey of the UK general public supports the principle of hospital triage based on survival probability along with other factors in the context of COVID-19 [44].

### Unanswered questions and future research

Whether robust study results will become available in the coming years remains to be seen. Especially when it comes to triage, many questions are still unanswered and are likely to remain so since it is almost impossible to meet design requirements for randomised trials on this subject.

## Data Availability

No datasets were generated or analysed during the current study.

## Abbreviations

adj.: Adjusted
ALS: Advanced life support
ASAV: Amberg-Schwandorf algorithm
AW: Airway
BCD: Battlefield casualty drills
BeS: Benzyl salicylate
CG: Control group
COT: Criteria Outcomes Tool
CS: Card-sort exercise
d: Days
DD: Dry decontamination
ED: Emergency department
EMS: Emergency medical services
FDNY: Fire Department of New York
FU: Follow-up
GAP: Glasgow-Age-Pressure Score
GCS: Glasgow Coma Scale
GPP: Good (clinical) practice point
h: Hours
IG: Intervention group
IQR: Interquartile range
ISS: Injury Severity Score
LoE: Level of evidence
LPS: Ladder pipe system
LS: Live simulation
m: Months
MCI: Mass casualty incident
MD: Mean difference
MeS: Methyl salicylate
MIMMS: Major Incident Medical Management and Support
MMS: Modified Military Sieve
MPTT: Modified Physiological Triage Tool
MS: Military Sieve
n.s.: Not significant
NARU: National Ambulance and Resilience Unit
NEWS2: National Early Warning Score 2
PPATS: Pediatric Physiological and Anatomical Triage Score
RAMP: Rapid Assessment of Mentation and Pulse
RCT: Randomised controlled trial
RoB: Risk of bias
RTS: Revised Trauma Score
s: Seconds
SBP: Systolic blood pressure
SD: Standard deviation
START: Simple Triage and Rapid Treatment
TASC: Tactical abbreviated surgical care
TD: Technical decontamination
TRTS: Triage Revised Trauma Score
TT: Triage trainer
VR: Virtual reality
y: Years
